# Supplementary material for: Different Mechanisms Cause Hypomethylation of Both H19 and KCNQ1OT1 Imprinted Differentially Methylated Regions in Two Cases of Silver–Russell Syndrome Spectrum
Source: Genes (Basel). 2022 Oct 16;13(10):1875. doi: 10.3390/genes13101875 (PMC9602374; doi:10.3390/genes13101875)
Supplement: Supplementary file 1 [file genes-13-01875-s001.zip › genes-1885075-supplementary.pdf]

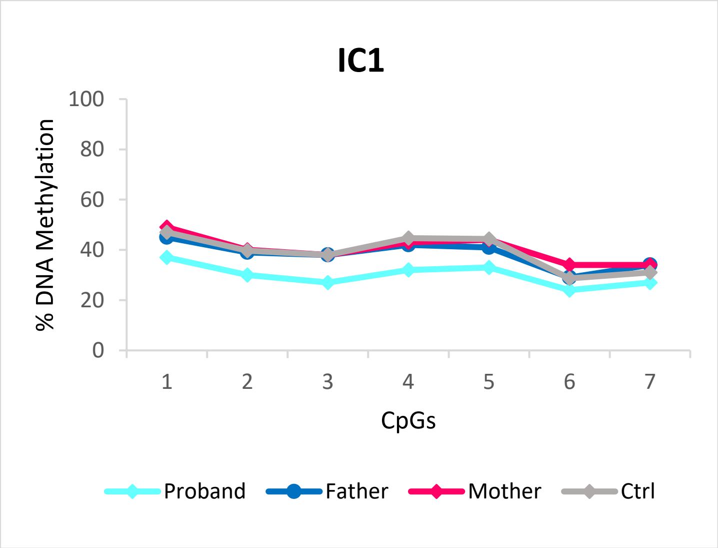
IC1
100
80
60
40
20
0
% DNA Methylation
1
2
3
4
5
6
7
CpGs
Proband
Father
Mother
Ctrl

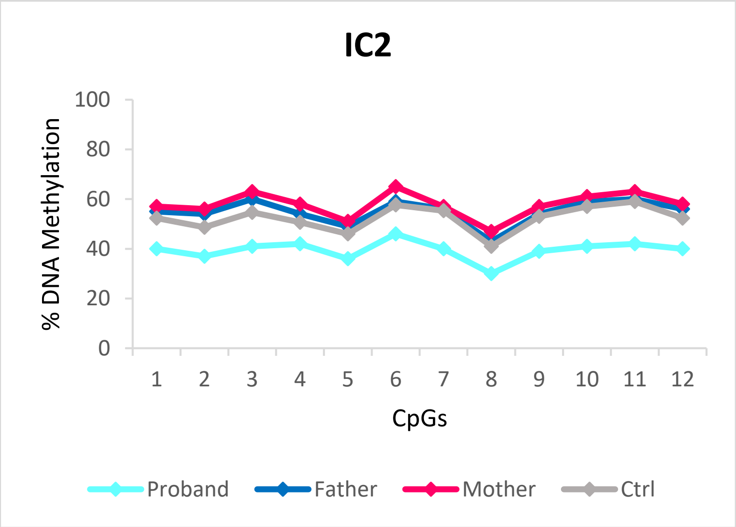
IC2
100
80
60
40
20
0
% DNA Methylation
1
2
3
4
5
6
7
8
9
10
11
12
CpGs
Proband
Father
Mother
Ctrl

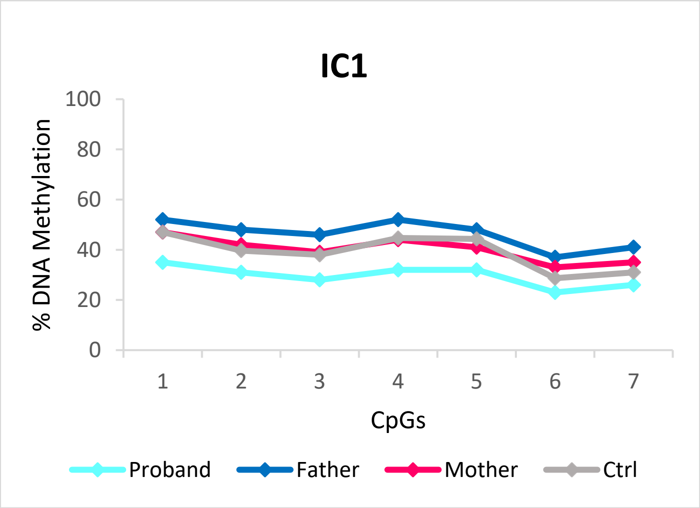
IC1
100
80
60
40
20
0
% DNA Methylation
1
2
3
4
5
6
7
CpGs
Proband
Father
Mother
Ctrl

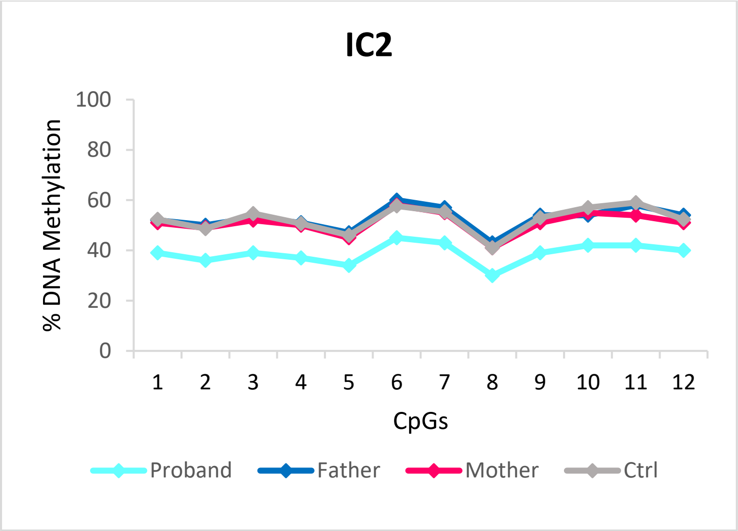

Figure S1. DNA methylation analysis by sodium bisulfite treatment and pyrosequencing of the 11p15.5 ICs of the probands and their parents. IC1 and IC2 methylation levels detected in the probands (light blu), their fathers (dark blue), their mothers (dark pink) and three unaffected individuals reported as average (grey). Mean methylation values of IC1 and IC2 were 30% and 39,5% in proband 1, 29,5% and 39 % in proband 2; 39% and 52% in controls average.

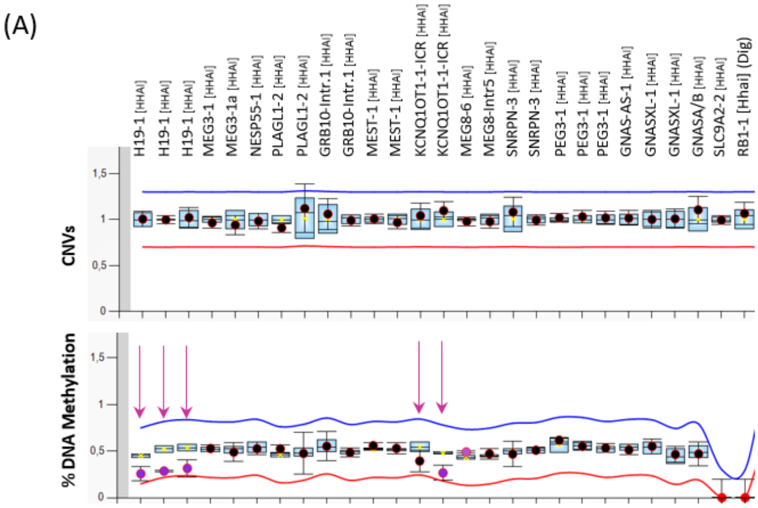

(B)

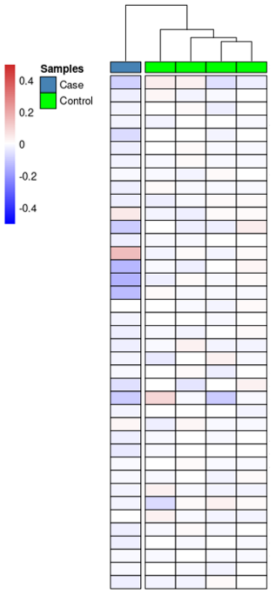

iDMRs	Proband 2	Controls average	Standard dev
PPIEL:Ex1-DMR	0,60	0,69	0,05
DIRAS3:Ex2-DMR	0,51	0,54	0,02
DIRAS3:TSS-DMR	0,55	0,56	0,02
ZDBF2/GPR1:IG-DMR	0,54	0,56	0,01
NAP1L5:TSS-DMR	0,50	0,57	0,01
FAM50B:TSS-DMR	0,53	0,56	0,01
PLAGL1:alt-TSS-DMR	0,53	0,54	0,01
GRB10:alt-TSS-DMR	0,52	0,53	0,01
PEG10:TSS-DMR	0,51	0,54	0,01
MEST:alt-TSS-DMR	0,56	0,59	0,02
HTR5A:TSS-DMR	0,64	0,59	0,02
ERLIN2:Int6-DMR	0,45	0,55	0,03
PEG13:TSS-DMR	0,58	0,60	0,01
INPP5F:Int2-DMR	0,85	0,70	0,02
H19/IGF2:IG-DMR	0,49	0,62	0,02
IGF2:Ex9-DMR	0,38	0,53	0,02
KCNQ1OT1:TSS-DMR	0,42	0,54	0,01
RB1:Int2-DMR	0,57	0,57	0,01
MEG3:TSS-DMR	0,57	0,58	0,00
MKRN3:TSS-DMR	0,83	0,85	0,01
MAGEL2:TSS-DMR	0,47	0,49	0,02
NDN:TSS-DMR	0,45	0,46	0,03
SNRPN:alt-TSS-DMR	0,62	0,62	0,02
SNRPN:Int1-DMR1	0,36	0,41	0,03
SNRPN:Int1-DMR2	0,50	0,59	0,08
SNURF:TSS-DMR	0,53	0,55	0,01
IGF1R:Int2-DMR	0,62	0,59	0,02
ZNF597:TSS-DMR	0,45	0,47	0,01
ZNF331:alt-TSS-DMR1	0,39	0,41	0,01
ZNF331:alt-TSS-DMR2	0,50	0,51	0,01
PEG3:TSS-DMR	0,57	0,58	0,01
MCTS2P:TSS-DMR	0,47	0,48	0,02
NNAT:TSS-DMR	0,72	0,73	0,05
L3MBTL1:alt-TSS-DMR	0,57	0,57	0,02
GNAS-NESP:TSS-DMR	0,54	0,56	0,01
GNAS-AS1:TSS-DMR	0,50	0,52	0,01
GNAS-XL:Ex1-DMR	0,52	0,53	0,01
GNAS-A/B:TSS-DMR	0,53	0,53	0,01
SNU13:alt-TSS-DMR	0,49	0,51	0,02

Figure S2. Extension of DNA methylation analysis at multiple imprinted loci by (A) MS-MLPA (MLID ME034 kit) and (B) methylome Illumina Epic array on the proband 2 DNA. In the table values exceeding +/- 3 standard deviation from average of controls are in blue (-) or red (+).
